# “I’m gonna lose my strength, I’m gonna seize and die, And all that Jazz”! Neurological diseases in jazz legends

**DOI:** 10.1590/0004-282X-ANP-2021-0067

**Published:** 2021-12-17

**Authors:** Francisco Manoel Branco Germiniani, Carlos Henrique Ferreira Camargo, Léo Coutinho, Hélio Afonso Ghizoni Teive

**Affiliations:** 1 Universidade Federal do Paraná, Hospital de Clínicas, Departamento de Medicina Interna, Serviço de Neurologia, Unidade de Distúrbios do Movimento, Curitiba PR, Brazil.; 2 Universidade Federal do Paraná, Hospital de Clínicas, Departamento de Medicina Interna, Programa de Pós-Graduação em Medicina Interna, Grupo de Doenças Neurodegenerativas, Curitiba PR, Brazil.

**Keywords:** History of Medicine, Nervous System Diseases, Music, História da Medicina, Doenças do Sistema Nervoso, Música

## Abstract

Even though jazz is a musical style that excels in improvisation and virtuosity, it is not without its share of anecdotes, drama, and downright tragedy, and the biographies of jazz musicians and their demise are fraught with ominous and dire straits. Unsurprisingly, some would develop chronic and fatal diseases. The neurological diseases that afflicted the following six composers and musicians, all of whom are considered jazz legends, are briefly discussed: Charles Mingus, diagnosed with amyotrophic lateral sclerosis; Lester Young and Charlie Parker, both diagnosed with neurosyphilis; Thelonius Monk, who had possible frontotemporal dementia; George Gershwin, who died as a result of brain glioma; and Cole Porter, who developed phantom limb pain following an amputation. The association of lifestyles, with drug abuse, particularly alcohol and heroin, in addition to great sexual promiscuity factors contributed to the development of a series of diseases such as syphilis. In addition, we also described some fatalities such as neurodegenerative diseases and cerebral glioma.

## RESUMO

Embora o *jazz* seja um estilo musical que prima pela improvisação e pelo virtuosismo, não é isento de drama e tragédia, e as biografias dos músicos de *jazz* e a sua morte estão repletas de dificuldades sinistras e terríveis. Alguns desenvolveriam doenças crônicas e fatais. São brevemente discutidas as doenças neurológicas que afligiram os seguintes seis compositores e músicos, todos eles considerados lendas do *jazz*: Charles Mingus, diagnosticado com esclerose lateral amiotrófica; Lester Young e Charlie Parker, ambos com neurosífilis; Thelonius Monk, que teve uma possível demência frontotemporal; George Gershwin, que morreu em decorrência de glioma cerebral; e Cole Porter, que desenvolveu dor de membro fantasma após uma amputação. A associação do estilo de vida com o abuso de drogas, particularmente álcool e heroína, além de promiscuidade sexual, contribuiu para o desenvolvimento de uma série de doenças, por exemplo, a sífilis. Também descrevemos algumas fatalidades, como doenças neurodegenerativas e glioma cerebral.

## INTRODUCTION

In the 19th century *fin-de-siècle* New Orleans, a new musical manifestation emerged: jazz music. This new phenomenon had its roots in the blues, a form of folk music created by African Americans, and ragtime, a black version of European piano music^1,2^.

Jazz would reach its heyday in the second half of the 20th century, initially in the USA. During this period, it existed in various forms and was being performed and written by great musicians and composers, some of whom became jazz legends^1,2,3^.

The purpose of this review was to briefly discuss the neurological diseases that affected a select group of jazz musicians and composers, some due to their lifestyles and some due to fatality.

## CHARLES MINGUS AND AMYOTROPHIC LATERAL SCLEROSIS

**Figure 1:**
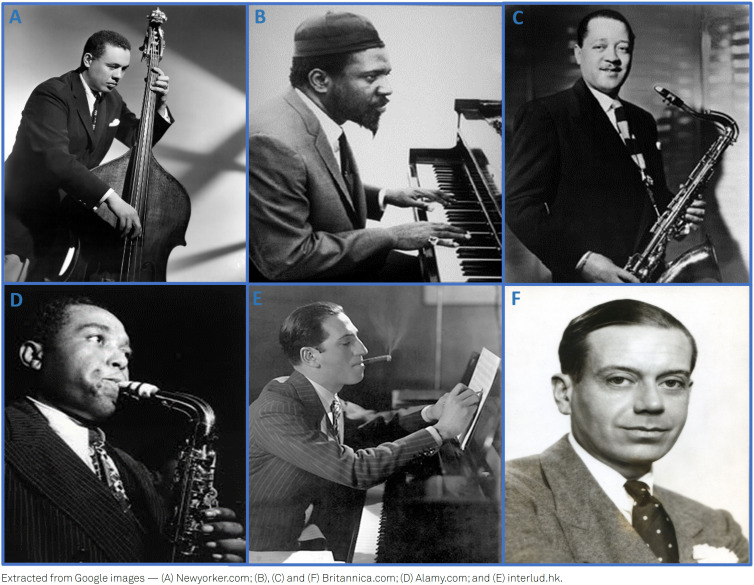
(A) Charles Mingus (1922–1979); (B) Thelonious Monk (1917–1982); (C) Lester Young (1909–1959); (D) Charlie Parker (1920–1955); (E) George Gershwin (1898–1937); (F) Cole Porter (1891–1964).

Charles Mingus (1922–1979) (Figure 1A) was a jazz composer and a gifted double bassist. During his career, he received distinctions from various institutions, such as the National Endowment for the Arts, the Smithsonian Institute, the Guggenheim Foundation, and Yale University^4,5,6^. In the 1970s, Mingus experienced progressive lower limb weakness associated with muscle atrophy; in 1977, the diagnosis was done for amyotrophic lateral sclerosis (ALS)^3,4,5,6^. He worsened progressively and used a wheelchair until his death in 1979^3,4,5,6^.

## THE ENIGMATIC DISEASE OF THELONIUS MONK

Thelonius Sphere Monk (1917–1982) (Figure 1B) was considered an innovator and the founder of bebop, a new type of jazz^6,7^. He was a frequent consumer of alcohol and hallucinogenic drugs, particularly heroin, leading to his arrest and banishment from performing in New York City for 6 years^3,6,7^. There is clear evidence that Monk had depressive behavior, developing progressive mental confusion intermingled with episodes of intense restlessness and excitement, followed by periods of depression, apathy, and mutism; in the 1960s, he was admitted to a psychiatric hospital in San Francisco, California^3,6,7^. He was diagnosed with “unclassified schizophrenia,” but his psychotic and cognitive conditions worsened, culminating in complete mutism^3,6,7^. In retrospect, the possible diagnoses could be bipolar disorder and frontotemporal dementia (FTD), starting with behavioral disorder followed by language disorder and subsequent dementia. Another possibility is cerebrovascular disease secondary to substance abuse^3,6,7^. In 1982, Monk suffered a stroke and died; the diagnosis with a ruptured cerebral aneurysm was questioned but never confirmed^3^.

## JAZZ MUSICIANS AND NEUROSYPHILIS

In 2017, Breitenfeld et al. retrospectively evaluated the diagnosis of neurosyphilis in about 1,500 composers and musicians, including many jazz artists.^8^ The authors concluded that Lester Willis Young and Charles “Bird” Parker had neurosyphilis^8^.

Lester “Prez” Young (1909–1959) (Figure 1C) was a jazz saxophonist, who became addicted to alcohol and other drugs and developed cirrhosis, culminating in acute upper gastrointestinal bleeding and his consequent death. Young also had a history of coronary insufficiency and cognitive impairment with confirmed neurosyphilis^3,6,8,9^.

Charles “Bird” Parker (1920–1955) (Figure 1D) was a jazz saxophonist who died very young as a result of acute pneumonia. He had a history of alcohol and heroin abuse with previous diagnoses of cirrhosis, upper gastrointestinal bleeding, and myocardial infarction^3,6,8,9,10^. Following a review of his medical records and based on the presence of behavioral and dementia disorders, as well as a positive Wasserman test, Parker was diagnosed with neurosyphilis^3,6,8,9,10^.

## GEORGE GERSHWIN AND BRAIN GLIOMA

George Gershwin (born Jakob Bruskin Gershovitz, 1898–1937) (Figure 1E) was a famous American composer^11,12,13,14,15^. In 1936, Gershwin started to present with several neurological symptoms and uncinate seizures (sudden episodes of a burning rubber smell followed by short episodes of “mental lapse”)^6,11,12,13,14,15^. As his condition progressed, Gershwin experienced severe headaches associated with episodes of dizziness and behavioral disorders, developing signs and symptoms of intracranial hypertension before going into coma^6,11,12,13,14,15^. After his admittance to a hospital in 1937, ancillary tests revealed a cystic tumor with a mural nodule extending deeply into brain tissue. Despite urgent neurosurgery, he died in the immediate postoperative period; neuropathology confirmed the diagnosis with glioblastoma multiforme^6,11,12,13,14,15^.

## COLE PORTER AND PHANTOM LIMB PAIN

Cole Porter (1891–1964) (Figure 1F) came from a very wealthy family and studied at Yale and Harvard^13,16^. He remains one of the most outstanding composers the USA has produced^6,13,16^. In 1937, Porter fell from a horse and fractured his both femurs, leading to bacterial infection and consequent osteomyelitis; despite 33 operations, his staphylococcal osteomyelitis chronified^6,13,16^. He abused alcohol and narcotics because of the chronic pain and, in 1958, his right lower limb was amputated. He subsequently began to experience pain in the amputated limb and was diagnosed with phantom limb pain^6,13,16^. Porter died in 1964 from chronic renal failure^3,13,16^.

## PSYCHIATRY, NEUROLOGY, AND MUSICIANS

Psychiatry in the days of these jazzmen was mainly asylum-centered^3^. Mentally ill patients were institutionalized for life, as therapeutic prospects were neglected; the epidemics of neurosyphilis and alcoholism contributed to an increase in the number of patients locked in these facilities. Academic advancements in the field of psychiatry occurred in this period. Psychopharmacology remained incipient, but synthesis and clinical application of several compounds, such as bromides (1857), chloral (1869), barbiturates (1903), antihistamines (1942), and lithium (1948), were described until the 1950s. Other unusual treatment options of the time included infecting patients with malaria to treat neurosyphilis and inducing insulin coma to treat schizophrenia. Although substance abuse (first opium, chloral, and barbiturates, and later heroin) presented a vertiginous increase during the 19th and 20th centuries, it was not recognized as a relevant public health issue^17^.

Tracing a parallel, the history of classical music presents many cases of neurological disease: neurosyphilis (Bedřich Smetana), ALS (Dmitri Shostakovich), stroke (Glenn Gould), aphasia (Vissarion Shebalin and Randall Thompson), Tourette’s syndrome (Amadeus Mozart), and dystonia (Robert Schumann, Leon Fleisher, and Gary Graffman)^18,19^.

It remains undisclosed if jazz musicians – or musicians in general – are more prone to neurological disease than the general population; their hedonistic lifestyle might have epigenetically contributed to genetically driven neurodegeneration.

In this historical review, the neurological diagnoses of six jazz composers and musicians were briefly discussed. The association of lifestyles, with drug abuse, particularly alcohol and heroin, in addition to great sexual promiscuity factors contributed to the development of a series of diseases, such as syphilis. In addition, we also described some fatalities: neurodegenerative diseases, such as ALS and frontotemporal dementia, and a case of cerebral glioma.
